# Lateral gene exchanges shape the genomes of amoeba-resisting microorganisms

**DOI:** 10.3389/fcimb.2012.00110

**Published:** 2012-08-21

**Authors:** Claire Bertelli, Gilbert Greub

**Affiliations:** Center for Research on Intracellular Bacteria, Institute of Microbiology, University Hospital Center and University of LausanneLausanne, Switzerland

**Keywords:** amoeba, intracellular bacteria, giant virus, gene transfer, evolution

## Abstract

Based on Darwin's concept of the tree of life, vertical inheritance was thought to be dominant, and mutations, deletions, and duplication were streaming the genomes of living organisms. In the current genomic era, increasing data indicated that both vertical and lateral gene inheritance interact in space and time to trigger genome evolution, particularly among microorganisms sharing a given ecological niche. As a paradigm to their diversity and their survival in a variety of cell types, intracellular microorganisms, and notably intracellular bacteria, were considered as less prone to lateral genetic exchanges. Such specialized microorganisms generally have a smaller gene repertoire because they do rely on their host's factors for some basic regulatory and metabolic functions. Here we review events of lateral gene transfer (LGT) that illustrate the genetic exchanges among intra-amoebal microorganisms or between the microorganism and its amoebal host. We tentatively investigate the functions of laterally transferred genes in the light of the interaction with their host as they should confer a selective advantage and success to the amoeba-resisting microorganisms (ARMs).

## Introduction

For many years following the publication of “*The origin of species*” by Charles Darwin (Darwin, 1859), the evolutionary history of the living organisms was represented by structures of trees that represent their common descent. However, this representation ignores the significance and the importance of LGT that allowed ancestral prokaryotes, and further on unicellular eukaryotes, to rapidly increase their genetic variability at a much faster rate than allowed by vertical inheritance, duplications and mutations (Keeling and Palmer, 2008; Lopez and Bapteste, 2009). It was progressively accepted that LGT have contributed to shape bacterial, archaeal, and eukaryotic genomes rending difficult to represent the vertical evolutionary history of these organisms (Andersson, 2005; Lopez and Bapteste, 2009; Olendzenski and Gogarten, 2009; Raoult, 2010b; Danchin and Rosso, 2012). From 1975, several new illustrations of evolution such as a “network-like representation,” a “reticulated tree,” or a “ring of life” have been proposed to account for the importance of horizontal transfers (Paz and Espinosa, 2010). More recently, to incorporate the theories of multiplicity and de-novo creation of genes, the “rhizome of life” was proposed as a representation of the evolution of species and the chimerism of bacterial genomes (Raoult, 2010b; Merhej et al., 2011).

In the light of the genome sequencing era, a growing number of whole genome analyses assessed the importance of lateral transfer in the constitution of gene repertoire (Doolittle, 1999; Doolittle and Bapteste, 2007) that reflects the organism lifestyle. Symbiotic and parasitic microorganisms, considered as extreme specialists, were shown to undergo genome reduction and have small gene repertoires due to their dependence on multiple host cell factors (McCutcheon and Moran, 2012). On the contrary, amoeba-resisting microorganisms (ARMs), which include both viruses and bacteria, often exhibit larger genomes than their mammalian-infecting relatives (Moliner et al., 2010). Amoebae, as a reservoir of numerous microorganisms sharing a sympatric lifestyle, i.e., microorganisms living in a community within amoebae, were proposed to bring these latter in close contact and facilitate genetic exchanges (Greub and Raoult, 2004; Moliner et al., 2010; Raoult, 2010a; Thomas and Greub, 2010). Indeed, microorganisms in large communities and sharing an ecological niche are more prone to genetic exchanges than isolated populations (Merhej et al., 2009; Raoult, 2010a). In this review, we summarize the recent findings on LGT in amoeba-infecting microorganisms highlighting the complex composite nature of their gene repertoire. Moreover, the function of exchanged genes is discussed in the context of symbiosis or host-pathogen interaction.

## Amoebae and their microbial hosts

### Amoebae as an evolutionary niche

The term free-living amoebae comprises more than 15,000 species (Adl et al., 2007) forming a heterogeneous group of phylogenetically distantly related protists that are widespread in water and soil ecosystems and display similar ecological characters. These unicellular eukaryotes were recently classified into two main suprakingdom-level groups; (i) the Excavata notably comprising the *Andalucia, Jakoba* (Jakobids), *Naegleria, Sawyeria, Vahlkampfia* (Heterolobosea) as well as the parasites *Trypanosoma, Leishmania* (Euglenozoa) and (ii) the Amoebozoa comprising among others *Acanthamoeba, Hartmannella, Vannella, Dictyostelium* and the medically important parasite *Entamoeba* (Hampl et al., 2009; Pawlowski and Burki, 2009).

Most amoebae live under the form of a trophozoite that replicates by binary fission, but in unfavorable conditions they can differentiate into a dormant form, the cyst. This latter is resistant to harsh conditions such as high temperature, desiccation, pH, and saline stress as well as disinfection processes (Thomas et al., 2004). Phagocytic amoebae graze on various microorganisms free-living or established in biofilms, including algae, bacteria, yeasts, and viruses (Rodriguez-Zaragoza, 1994). Microorganisms are phagocytosed and normally follow the endocytic pathway to be degraded in acidic phagolysosomes by a number of hydrolases (Greub and Raoult, 2004). However, several giant viruses and bacteria have evolved strategies to escape degradation, hence their naming as ARMs. They live symbiotically within their host or replicate in vacuoles before lysing the amoeba.

Taking profit from these characteristics, Rowbotham (1983) used cultures of amoebal cells to grow *Legionella* species. Since then, amoebal co-culture has become a method of choice to retrieve new microorganisms able to resist and grow in these professional phagocytes. This method uses amoebae as a cell background to inoculate environmental or medical samples, in order to retrieve ARMs (Lienard and Greub, 2011). However, the almost uniform use of *Acanthamoeba castellanii* (Thomas et al., 2006; Corsaro et al., 2009) and *A. polyphaga* (Greub et al., 2004b; Pagnier et al., 2008) largely biases and underestimates the diversity of known ARMs. Diversifying the species of amoeba used in co-culture experiments is required to improve our understanding of the pool of amoebal symbionts and parasites.

**Figure 1:**
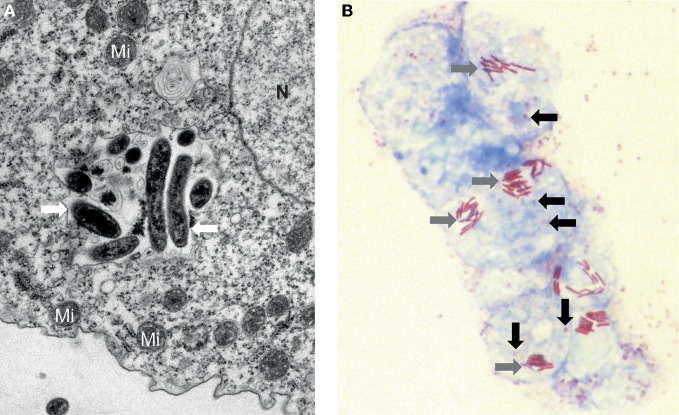
**Amoebae may hide several ARMs**. **(A)** Electron microscopy of an inclusion containing a mixed population of microorganisms (white arrow) recovered from a water-humidifier co-cultured in an amoeba of the species *Acanthamoeba castellanii*. Mi: Mitochondrion, N: Nucleus. Magnification 10,000×. **(B)** Photonic microscopy of *Legionella spp.* and *Lausannevirus* in *A. castellanii.* Several amoebae contain simultaneously both the giant virus (black arrow) and the rod-shaped bacteria *Legionella* (grey arrow). Gimenez staining, Magnification 1000x.

Concisely, amoebae can act as a replicative niche and a reservoir of ARMs that are established in water and soil environments (Greub and Raoult, 2004). As shown in Figure 1, amoebae may hide several ARMs in their cytoplasm or more commonly in phagocytic vacuoles. The cyst may function as an armor to protect internalized microorganism from difficult external conditions as well as disinfection procedures. Moreover, the development of strategies to resist microbicidal effectors by ARMs may help selecting virulence traits enabling to survive in the macrophages, the first line of human defense, as it is the case for *Mycobacteria*, *Legionella*, *Parachlamydia* (Greub, 2009; Lamoth and Greub, 2009; Salah et al., 2009), and Mimivirus (Ghigo et al., 2008). More importantly, amoebae were lately suggested as a place that favor genetic exchanges by bringing in close vicinity ARMs (Moliner et al., 2010; Thomas and Greub, 2010; Merhej et al., 2011). The recent sequencing of some amoebal genomes such as *Entamoeba histolytica, E. dispar, Dictyostelium discoideum*, and *A. castellanii* provide the opportunity to highlight the first hints on the genetic exchanges between the amoebae and their intracellular microbes.

### Amoeba-resisting viruses

The search for amoeba-resisting viruses (ARVs), i.e., viruses able to replicate alone or in combination with others within amoeba, started with the discovery and the sequencing of Mimivirus in the early 2000s (La Scola et al., 2003; Raoult et al., 2004), which raised an extraordinary interest. Within less than a decade, the known complexity of ARVs was boosted by (i) the discovery of the small virophage Sputnik (La Scola et al., 2008), (ii) the description of Marseillevirus and Lausannevirus, two large viruses encoding histone-like proteins (Boyer et al., 2009; Thomas et al., 2011), as well as (iii) the publication of *Megavirus chilensis* (Fischer et al., 2010; Arslan et al., 2011), the largest identified virus harboring a 1.26 Mb genome (Table 1). According to new systematic searches, ARVs seem to be fairly common in the environment (La Scola et al., 2010).

**Table 1:** **Amoeba-resisting viruses with a publicly available genome sequence**.

**Family**	**Microorganism**	**Genome**	**Host**	**References**
Mimiviridae	Mimivirus	1.18 Mb	*Acanthamoeba*	Raoult et al., 2004
Mimiviridae	Megavirus chilensis	1.26 Mb	*Acanthamoeba*	Arslan et al., 2011
Mimiviridae^*^	*Cafeteria roenbergensis* virus	0.730 Mb	*C. roenbergensis*	Fischer et al., 2010
–	Sputnik	0.018 Mb	*Acanthamoeba*	La Scola et al., 2008
Marseilleviridae	Marseillevirus	0.368 Mb	*Acanthamoeba*	Boyer et al., 2009
Marseilleviridae	Lausannevirus	0.346 Mb	*Acanthamoeba*	Thomas et al., 2011

* Classification proposed by Colson et al. (2011).

Most ARVs possess pseudo-icosahedral capsids hiding intricate large dsDNA genomes that pushed forward the recognized limits of viral genome size. Therefore, they were called (i) “giant viruses” (La Scola et al., 2003; Raoult et al., 2004), a concept previously used for large algal DNA viruses (Van Etten and Meints, 1999), or (ii) “giruses” (Legendre et al., 2012). Another interesting girus is *Cafeteria roenbergensis* virus (CroV) that infects a marine phagocytic flagellate belonging to the *Chromalveolata* (Fischer et al., 2010). CroV was not demonstrated to replicate in amoebae, but it is worth discussing as it shares many similarities with other ARVs. At the opposite, Sputnik is a 18 kb virophage able to replicate in *A. castellanii* only when Mimivirus, or its close relative Mamavirus, co-infects the amoeba (La Scola et al., 2008). Sequences homologous to Sputnik were detected in the environmental dataset of the Global Ocean Survey, suggesting that it may represents a new virus family, but its classification is currently unclear.

Mimivirus, Megavirus, CroV, Marseillevirus, and Lausannevirus belong to the monophyletic class of nucleo-cytoplasmic large DNA virus (NCLDV) (Iyer et al., 2006). They were classified into two main families of NCLDV, the Mimiviridae and the Marseilleviridae (Table 1). NCLDVs only share a core genome of 30–47 genes (Iyer et al., 2006; Yutin et al., 2009). Thus, core genes only represent a minor fraction of the gene repertoire, whereas ORFans, i.e., genes that do not have homologs in other organisms, and dispensable genes, i.e., genes present in two or more NCLDVs, are the major constituent of these viral genomes.

The reconstruction of deep phylogenetic relationship from viral sequences is controversial because of the rapid evolutionary rate of viruses and the presence of numerous horizontal transfers (Moreira and Brochier-Armanet, 2008), in particular from host genomes. Nevertheless, several evidences suggest that these giruses have evolved from a common ancestor. The two families of distant giruses, *Marseilleviridae* and *Mimiviridae*, harbor an unusual genomic repertoire that includes genes for protein translation, a hallmark of cellular organisms (Raoult et al., 2004; Boyer et al., 2009; Arslan et al., 2011). Moreover, the presence of tRNA synthetases in some viruses and their absence in others supports the idea that all *Mimiviridae* evolved by reductive evolution from a common ancestor, potentially a cellular ancestor: four tRNA synthetases homologs have been found in Mimivirus and Megavirus, an additional one in both CroV and Megavirus, and two additional ones are present in Megavirus only (Ghigo et al., 2008). A further example of lineage-specific deletion is given by the DNA photolyase: CroV possesses two intact copies, Mimivirus harbors fragmented ortholog remnant of one of them, and finally Megavirus encodes one intact ortholog and one ortholog split in two parts by a transposase (Ghigo et al., 2008).

The amazing diversity in genome size and gene repertoire among these phylogenetically related viruses questions the respective importance of both LGTs and vertical inheritance in evolution. The large differences observed in genome size (0.018–1.3 Mb) would imply either an extensive genome growth via LGTs or a divergent reductive evolution in the different phyla. An increased propensity to acquire genes of foreign origin surely accounts for such differences in genome size (Monier et al., 2007) and some authors even consider viruses as “bags of genes” (Hendrix et al., 2000; Moreira and Lopez-Garcia, 2005). However, it is questionable whereas LGTs are sufficient to explain such a large variation. Moreover, core genes seem to have originated from different kingdom, including eukaryotes, bacteria, and bacteriophages (Koonin and Yutin, 2010). These observations are in agreement with the scenario of a bacteriophagic origin of NCLDV (Koonin and Yutin, 2010). At this stage, a mix of genes from very different eukaryotic and bacterial organisms were acquired concurrently to the loss of phage genes except those essential for genome replication and virion formation (Koonin and Yutin, 2010). The following section attempts to provide an overview of the extent of genetic exchanges documented in ARVs.

### Amoeba-resisting bacteria

**Figure 2:**
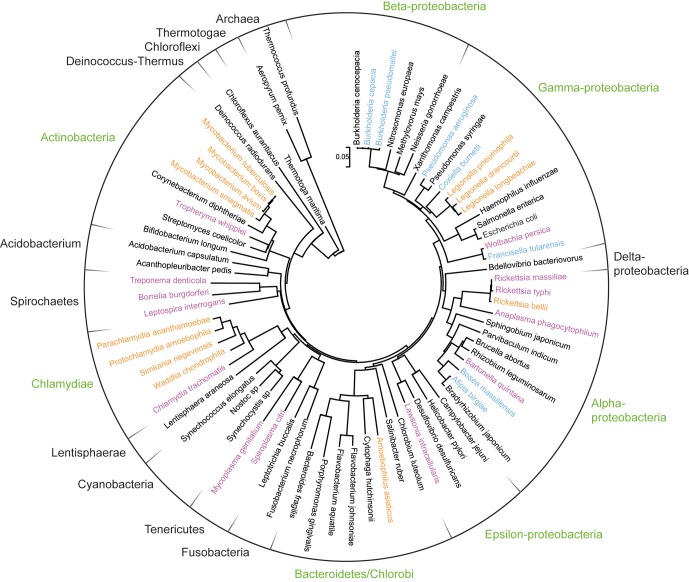
**Phylogenetic positioning of amoeba-resisting bacteria**. The ribosomal RNA small subunit (16S) of Bacteria and two Archaea was aligned with Muscle and a neighbor-joining tree was subsequently built with Mega 5 (complete deletion, gamma distributed). The tree was rooted between Archaea and Bacteria. Bacterial phyla that comprise amoeba-resisting bacteria are indicated in green. Bacteria isolated from amoeba or growing within amoeba are highlighted in orange whereas those shown to resist amoebal phagocytosis *in vitro* are shown in blue. Finally other intracellular and fastidious bacteria are shown in purple.

A large variety of amoeba-resisting bacteria (ARBs) have been isolated using amoebal co-culture or directly retrieved from their host by amoebal enrichment (Greub et al., 2004b; Horn and Wagner, 2004; Lienard and Greub, 2011). In addition, many microorganisms have been shown to survive *in vitro* in amoebae such as different *Burkholderia, Coxiella burnetii*, a strain of *E. coli*, *Francisella tularensis, Helicobacter pylori, Listeria monocytogenes, Porphyromonas gingivalis*, and *Vibrio cholerae* (Greub and Raoult, 2004; Wagner et al., 2006). As shown in Figure 2, these encompass various clades scattered through the prokaryotic phylogeny, including members of the *Actinobacteria, Bacteroidetes, Chlamydiales, Firmicutes*, and different subdivisions of *Proteobacteria* (α, β, γ, ε). Although ARBs are found in most major taxonomic phyla, only few major groups of bacteria have been studied more extensively, including *Mycobacteria*, *Chlamydia*-related bacteria, *Rickettsia* and *Legionellae* and will be the focus of this review (Table 2).

**Table 2:** **Selected amoeba-resisting bacteria with a publicly available genome sequence**.

**Classification**	**Microorganism**	**Genome**	**Host**	**References**
*Actinobacteria*	*Mycobacterium avium*	4.83 Mb	*Acanthamoeba*	Li et al., 2005
	*Mycobacterium marinum*	6.64 Mb	*Acanthamoeba*	Stinear et al., 2008
	*Mycobacterium smegmatis*	6.99 Mb	*Acanthamoeba*	Fleischmann et al., 2006
	*Mycobacterium tuberculosis*	4.41 Mb	*Acanthamoeba*	Cole et al., 1998
*Bacteroidetes*	*Amoebophilus asiaticus*	1.88 Mb	*Acanthamoeba*	Schmitz-Esser et al., 2010
	*Porphyromonas gingivalis*	2.34 Mb	*A. castellanii*^a^	Nelson et al., 2003
	*Flavobacterium johnsoniae*	6.10 Mb	*A. polyphaga*^a^	McBride et al., 2009
*Chlamydiales*	*Parachlamydia acanthamoebae*	3.07 Mb	*A. castellanii*	Collingro et al., 2011
	*Protochlamydia amoebophila*	2.41 Mb	*A. castellanii*	Horn et al., 2004
	*Simkania negevensis*	2.50 Mb	*A. castellanii*^a^	Collingro et al., 2011
	*Waddlia chondrophila*	2.12 Mb	*A. castellanii*	Bertelli et al., 2010
α-proteobacteria	*Rickettsia bellii*	1.52 Mb	*A. polyphaga*	Ogata et al., 2006
	*Odyssella thessalonicensis*	2.85 Mb^b^	*Acanthamoeba*	Georgiades et al., 2011
γ-proteobacteria	*Legionella drancourtii*	4.16 Mb^b^	*A. polyphaga*	Gimenez et al., 2011
	*Legionella longbeachae*	4.08 Mb	*Acanthamoeba*	Kozak et al., 2010
	*Legionella pneumophila*	3.50 Mb	*Acanthamoeba*	Cazalet et al., 2004; Chien et al., 2004

a Shown to grow in amoebae, but maybe not the natural host.

b Unfinished genome sequence.

***Actinobacteria:*** The genus *Mycobacteria* comprises many bacteria such as *M. tuberculosis* and *M. leprae* that are major threat to human health and 19 different species have been sequenced to date. Most *Mycobacteria*, including members of the nontuberculous and the tuberculous complex groups, have been shown to survive and grow in various amoebae such as *Acanthamoeba sp., Dictyostelium discoideum*, and *Tetrahymena pyriformis* (Thomas and McDonnell, 2007; Mba Medie et al., 2011). It was suggested that adaptations for the intra-amoebal survival and multiplication may have facilitated the virulence toward mammalian cells (Molmeret et al., 2005).

***Chlamydiales:*** The *Chlamydiales* order includes important human and animal pathogens such as *Chlamydia trachomatis*. A large number of amoebal symbionts called “*Chlamydia*-related bacteria” have been retrieved and shown to infect various organisms, including free-living amoebae, arthropods, insects as well as vertebrates (Corsaro and Greub, 2006; Horn, 2008). Four of them have been fully sequenced (Table 2), two additional strains have been published as draft genomes and new representative of the *Parachlamydiaceae* and *Criblamydiaceae* families are currently being sequenced (Horn et al., 2004; Greub et al., 2009; Bertelli et al., 2010; Collingro et al., 2011).

***Alpha-proteobacteria:*** Highly pathogenic representatives of *Rickettsia* are typically transmitted through arthropods (Merhej and Raoult, 2011) but some *Rickettsia*-like endosymbionts were observed in *Acanthamoeba* (Fritsche et al., 1999). *Rickettsia bellii*, a species that diverged early in evolution, was shown to survive for three weeks in *A. polyphaga* (Ogata et al., 2006). *Odyssella thessalonicensis* is a strict intracellular bacteria isolated from an air conditioning system in Greece (Doolittle and Bapteste, 2007). An unfinished genome sequence has been released, but unfortunately, little information is known on the genetic characteristics of this bacterium.

***Gamma-proteobacteria:*** *Legionellae* are distributed worldwide and commonly found in water environments where they are major components of the biofilms (Rogers et al., 1994). Since the first culture of *L. pneumophila*, the causative agent of the Legionnaire's disease, within amoeba (Rowbotham, 1980), more than fifty strains of *Legionella* have been discovered. Although they are sometimes considered as all growing in amoebae, only a few have been shown to grow within amoebae (Rowbotham, 1980; Neumeister et al., 1997; La Scola et al., 2004) and the genomes of three species have been sequenced.

#### Geographic distribution and biology

ARBs are distributed worldwide and have been principally isolated from samples of aquatic environments where they are predominantly found in biofilms. The numerous and diverse bacteria entertain close relationships in biofilms where conjugation and transformation occur frequently enabling genetic exchanges (Molin and Tolker-Nielsen, 2003). The current explosion of new genome sequences significantly contributed to understand the mechanisms underlying genome evolution of the ARBs (Darby et al., 2007; Horn, 2008; Moran et al., 2008; Moya et al., 2008) and should enable us to conduct more extensive studies on genetic exchanges between ARMs, their biology and their interactions with the host cell. Indeed, ARBs show major differences in the mechanisms of host cell interaction. As an example, they have developed numerous ways to escape phagocytosis in amoebae: some escape the phagocytic pathway and replicate within the host cell cytoplasm (Birtles et al., 2000; Horn et al., 2001) whereas others block the maturation of phagolysosomes at various stages and replicate in host-derived vacuoles (Greub and Raoult, 2002; Isberg et al., 2009).

#### Genomic features

All the aforementioned ARBs share some similarities in their genomic characteristics. It has been shown that free-living bacteria tend to have larger genomes than the intracellular specialists that undergo genome reduction (Merhej et al., 2009). Although they are considered as specialized bacteria, ARBs harbor larger genomes compared to related organisms infecting human or other vertebrates (Moliner et al., 2010). For example, *Chlamydia*-related bacteria have genomes ranging from 2.1 to 3 Mb, i.e., twice two three times larger than classical *Chlamydiae*.

The genomes of *Rickettsia* and *Chlamydia*-related bacteria appear to be extensively shuffled by rearrangements, insertions and deletions and thus exhibit a very limited colinearity compared to related genomes (Ogata et al., 2006; Bertelli et al., 2010; Collingro et al., 2011). However, the signal may be dispersed by (i) the large difference in size that mimics numerous large insertions and (ii) the fact that these bacteria are more distantly related to one another than the closest relatives among them. To corroborate this hypothesis, the four strain of *L. pneumophila* fully sequenced to date are highly conserved with a single inversion of 250 kb taking place in strain Lens compared to the three other strains (Gomez-Valero et al., 2009).

As expected for strictly intracellular bacteria, they overall lack several global pathways or key enzymes for the synthesis of nucleotides, amino-acids, and cofactors (Chien et al., 2004; Horn et al., 2004; Bertelli et al., 2010; Schmitz-Esser et al., 2010; Collingro et al., 2011). In *Chlamydiales*, the pattern of missing pathways varies in each organism suggesting that these organisms have evolved by reductive evolution from an ancestor with enhanced if not complete biosynthetic capabilities. Interestingly, although *L. pneumophila* is known to be auxotrophic for several amino acids such as cysteine, methionine, phenylalanine, and tyrosine, genes necessary for their biosynthesis were found (Chien et al., 2004). On the contrary, key features for the host pathogen interaction are highly conserved, as for example the complete type III secretion system of *Chlamydia* or the type IV Dot/Icm system in *Legionella* used to translocate effectors into the host cell (Bertelli et al., 2010; Moliner et al., 2010).

In summary, ARBs exhibit interesting genome characteristics compared to related microorganisms that do not infect amoebae: larger genomes, more genetic rearrangements, and more extensive metabolic capabilities. Globally, most ARBs harbor a largely diverse gene repertoire that indicates the occurrence of numerous lateral gene transfers (LGTs) detailed below.

## Genetic exchanges

### Mechanisms of gene transfer

Several mechanisms allow genetic exchanges among organisms from the various domains of life (Paz and Espinosa, 2010). Briefly, virus-mediated transfers occur via transduction from phages (prokaryotes) or transposon (prokaryotes and eukaryotes). Prokaryotes use transformation, i.e., DNA uptake from the environment, or conjugation, i.e., DNA exchange through pilus-like systems, to transfer gene fragments or plasmids. Prokaryote to eukaryote transfers may arise as the result of the ingestion of cells, a process called phagotrophism, or symbiogenesis, i.e., the establishment of a permanent association.

Such events were notably involved in the formation of the animal lineage through the primary symbiosis forming the primary eukaryotic cell. Some authors hypothesized that Archaebacteria and an Actinobacteria interacted in the primary symbiotic event leading to the creation of mitochondria, a hypothesis that was soon rejected (Cavalier-Smith, 2002). Others suggested that an archaebacteria was invaded by a second prokaryote related to *α-proteobacteria* that had a bacteriovory ability similar to *Bdellovibrio* (Davidov et al., 2006; Cox et al., 2008). Georgiades et al. (2011) proposed that mitochondria are more related to *Rickettsiales* and the bacterium *Pelibacter ubique*. Recently, it was also noticed that mitochondria may be sister to *Rhizobiales, Rhodobacterales*, or *Rickettsiales* suggesting an eventual chimeric origin of mitochondria (Atteia et al., 2009; Abhishek et al., 2011; Georgiades and Raoult, 2011). Similarly, the symbiosis of a cyanobacterium with this primary eukaryotic cell was at the basis of the establishment of the chloroplast 1.2 billion years ago (Dyall et al., 2004). However, some lateral transfer events or chimeric events may have occurred with an ancestral *Chlamydiales*, as suggested by the high proportion of plant-like genes in chlamydiae (Brinkman et al., 2002).

Lateral transfers occur with varying frequency, magnitude and resulting fitness, which modulates the establishment of the transferred domain in the population. LGTs are frequent in prokaryotes and transferred genes can become integrated rapidly in the population thanks to short generation times (Paz and Espinosa, 2010). On the contrary, although increasingly reported, gene transfers involving eukaryotic species are still currently underrepresented in the litterature (Keeling and Palmer, 2008; Andersson, 2009). This is in part due to the large number of available prokaryotic genome sequence facilitating such analyses, compared to the currently restricted number of published sequence of unicellular eukaryotes.

### Mobile genetic elements

Mobile genetic elements (MGEs) are essential actors and markers of non vertical genome evolution (Frost et al., 2005). Indeed, they often encode mechanisms to spread in new host and form sites for the preferential acquisition of exogenic sequences. Insertions sequences (IS) and transposases, mobile endonucleases of the HNH family and DNA methylases that may form restriction/modification systems, as well as inteins are typical examples of MGEs commonly found in bacteria and archaea. As such, they were found in several, if not all, ARBs and we only provide a few examples below. Interestingly, they were also identified in several ARVs and we report them here in more details.

The genome sequence of *M. tuberculosis* harbor many IS (*n* = 54) that are preferentially integrated in intergenic regions, close to tRNAs (Cole et al., 1998), whereas *M. smegmatis* and *M. marinum* present fewer IS (Stinear et al., 2008). Similarly, *Chlamydia*-related bacteria all encode numerous entire or remnants of transposases, whereas their relatives *Chlamydia* have little if no trace of invasion by such mobile elements (Bertelli et al., 2010). Among ARBs, *A. asiaticus* is an interesting case as it exhibits a massive proliferation of MGEs (up to 24% of the genome), including abundant IS, but its genome seems relatively stable (Schmitz-Esser et al., 2010). An interesting example of composite transposon is found in *A. asiaticus* where the cluster of gene for lasso peptide synthesis is flanked by two IS, suggesting its ability to be mobilized for genetic transfer (Schmitz-Esser et al., 2010). The genomes of *Legionellae* exhibited a large plasticity indicated by the presence of numerous mobile elements and three plasmids in *L. pneumophila* (Chien et al., 2004; Gomez-Valero et al., 2009; Cazalet et al., 2010).

HNH and restriction-like endonucleases have been found in Marseillevirus (*n* = 10), Lausannevirus (*n* = 8), Megavirus (*n* = 6) and Mimivirus (*n* = 3) (Raoult et al., 2004; Boyer et al., 2009; Arslan et al., 2011; Thomas et al., 2011). In addition, a phage-type endonuclease discovered in Megavirus and two HNH flanking a prophage gene in Mimivirus might have been laterally acquired from prophage genomes.

Inteins are typical selfish elements that mediate protein splicing to trigger their excision from the protein precursor (Perler et al., 1994). They often contain a homing endonuclease (HE) that cuts double-stranded genomic DNA to integrate itself in the corresponding gene of non-infected organisms (Gogarten et al., 2002). Giruses are invaded by inteins of different types and at different locations (Table 3).

**Table 3:** **Inteins and their homing endonucleases in ARVs**.

**Microorganism**	**Infected gene**	**Intein insertion site**	Homing endonuclease^*^
Mimivirus	DNA polymerase B	Tli Pol-2	Complete
Megavirus	DNA polymerase B	Tli Pol	n.a.
Megavirus	DNA-directed RNA polymerase beta subunit	RPB2	n.a.
CroV	DNA polymerase B	Tli Pol-2	Remnant
CroV	Ribonucleoside-diphosphate reductase, alpha subunit	RIR1-h	Remnant
CroV	DNA-directed RNA polymerase beta subunit	RPB2-c	None
CroV	DNA Topoisomerase IIA	Top2-a	Complete
Lausannevirus	D6/D11-like helicase	-	Complete
Lausannevirus	Ribonucleoside- diphosphate reductase, alpha subunit	RIR1	Remnant
Marseillevirus	D6/D11-like helicase	-	Complete
Marseillevirus	Ribonucleoside-diphosphate reductase, alpha subunit	RIR1	Remnant

* Remnant is indicated if one or several conserved blocks (C-D-E-H) of the homing endonuclease is missing.

“n.a.” Information not available.

The eukaryotic-like DNA polymerase B of Mimivirus encodes an intein and its likely functional HE that is most closely related to extremophile archaea (Ogata et al., 2005a). Marseillevirus and Lausannevirus present two highly similar inteins and their HE in orthologous genes, suggesting that these MGEs were acquired by their ancestor and are now evolving differently toward degradation (Thomas et al., 2011). Megavirus and CroV also possess respectively two and four inteins that were until now not studied in detail. As it is often the case, all viral inteins are integrated in highly conserved genes related to replication, transcription, or DNA metabolism. These conserved genes constitutes preferential target as they are essential to the virus. These observations are in agreement with the hypothesis that viruses might play a central role in the transmission of inteins across species (Pietrokovski, 1998) and suggest that these mobile elements had or still have the ability to excise, spread an insert into new hosts. However, since the various ARVs present inteins in orthologous genes, further studies are required to investigate if these MGEs have spread in giruses sharing a sympatric lifestyle or if they were acquired by common ancestors.

The role and the exact origin of these MGEs in amoeba-infecting viruses are in most cases unclear. MGEs are often colocalized with genes of putative bacterial origin (Filee and Chandler, 2010). Like in bacteria, MGEs could thus be involved in promoting or facilitating lateral transfers, which may in turn provide some selective advantage to the ARVs by facilitating the acquisition of new advantageous genes.

### Extent of genetic exchanges in ARVs

The role of genetic exchanges has been a matter of intense debate since the publication of the Mimivirus (Raoult et al., 2004; Moreira and Lopez-Garcia, 2005; Ogata et al., 2005b). The first analyses suggested massive LGT from various origins, including members of the four domains of life. It was suggested that the genomes of giant viruses infecting protists are largely affected by LGTs and non-orthologous gene displacements (Filee et al., 2007; Fischer et al., 2010; Koonin and Yutin, 2010). Some authors even regarded these viruses as “bags of genes” thus suggesting that the amount of genes transferred from amoebae to viruses overweight the flux of genes in the opposite direction (Moreira and Lopez-Garcia, 2005; Moreira and Brochier-Armanet, 2008). It was shown that genes with an anomalous nucleotide composition may lead to the overestimation of LGTs (Monier et al., 2007), and family specific genes do not present an accelerated evolutionary rate and were not laterally exchanged (Ogata and Claverie, 2007). Several studies thus used BLAST-based searches and phylogenetic methods to investigate the occurrence of lateral transfers from host to virus, virus to host as well as between viruses and other microorganisms (Table 4). Unfortunately, the use of different cutoffs renders some results difficult to compare.

**Table 4:** **Events of lateral gene transfer with amoeba-resisting viruses**.

**Microorganism**	**Viruses^a^**		**Host**		**Eukaryota**		**Bacteria**		**Archaea**	
	**B**	**P**	**B**	**P**	**B**	**P**	**B**	**P**	**B**	**P**
Mimivirus	45	4	5	12–13	30	60	96	29	n.a.	1
Marseillevirus	59	51	n.a.	25	70	85	57	49	2	n.a.
Lausannevirus	2	n.a.	1	n.a.	2	n.a.	7	n.a.	-	n.a.

The table present the number of genes potentially laterally transferred as retrieved by BLAST-based (B) and by phylogeny-based (P) methods.

a In Megavirus, 44 genes were reported to match against non-viral sequences in the nr database, but no information on taxonomic classification is available.

#### Lateral gene transfers with eukaryotes

The increasing availability of genome sequences from viral host enabled to investigate the occurrence of LGTs between NCLDVs and eukaryotes, and more specifically their host, using BLAST-based searches (Filee et al., 2008). Mimivirus had the lowest proportion of genes (3%, *n* = 30) from potential eukaryotic origin. A preliminary analysis of Megavirus, the only other sequenced representative of the Mimiviridae, showed that only 17% of the 258 genes of Megavirus with no obvious homolog in Mimivirus match against the nr database (Arslan et al., 2011). However, the authors report no special affinity with potential donors and do not report any information on the taxonomic classification these donors. By contrast, Marseillevirus showed the highest propensity (*n* = 24, 5.6%) to acquire genes from its host (Boyer et al., 2010; Filee and Chandler, 2010). These results challenge the theory of the viruses as “bags of genes” that would have gained genomic content by acquiring genes from diverse sources since the divergence of the last common NCLDV ancestor. In this case, we would expect larger genomes to have acquired more genes by LGTs, which is not observed here. On another hand, some virus families may have gained over time a higher tendency to acquire genes, which would bias the estimation of LGT propensity.

Potential gene transfers were not extensively studied for Lausannevirus, but its ubiquitin encoding gene was shown to present a best hit against *A. castellanii*, suggesting a transfer between the virus and its host (Thomas et al., 2011). By comparing Mimivirus and *Entamoeba histolytica*, 5 genes (5%) were potentially acquired from its amoebal host, providing that the *Entamoeba* genome is representative of the natural host, *A. castellanii*, whose genome was not available at that time (Andersson, 2009). Finally, Colson et al. (Colson et al., 2011) reported that *A. castellanii* encodes a homolog to the major capsid of CroV. They hypothesized that *A. castellanii* might represent an ancient host for CroV itself or for its ancestor which then specialized to infect flagellate protists such as *Cafeteria roenbergensis.*

In Mimivirus, phylogenetic analyses confirmed the limited number of LGTs with eukaryotes (*n* = 60, 6%) and with its host (*n* is unkown, ~10% of 126 ORFs with eukaryotic homologs) (Moreira and Brochier-Armanet, 2008). However, these numbers do not take into account genes shared only by the virus and its amoebal host, rending impossible the phylogenetic reconstruction of an evolutionary history. In addition, Moreira et al. reported a few phylogenies supporting a transfer with the amoebae *Naegleria* and *Sawyeria*. Interestingly, large virus-like organisms that might be related to Mimiviridae were described in the cytoplasm of such amoebae (Schuster and Dunnebacke, 1974).

The histone-like proteins of Marseillevirus and Lausannevirus represent another intriguing case of potential lateral transfer. Heliothis zea virus, Bracovirus, and the ostreid herpesvirus were already shown to encode histone-like proteins probably acquired from their hosts (Cheng et al., 2002; Gad and Kim, 2008; De Souza et al., 2010). However, both Marseilleviridae harbor orthologs of as many as three histone-like proteins, two of them encoding for histone doublets, the third one being the fusion of a histone fold with an unknown domain (Thomas et al., 2011). A phylogenetic reconstruction clustered the various histone domains with different eukaryotic and archaeal histones, thus raising questions on their origin and their potential acquisition in a single or multiple events of lateral transfer. The function of these histone-like proteins has not been clearly shown yet. These histones were detected in the viral particle where they may help packaging DNA (Boyer et al., 2009), but they may as well have a role in modifying the chromatin structure of the host genome, a hypothesis that remains to be tested.

#### Lateral gene transfers with prokaryotes

Only few genes show some evidence of lateral transfer with archaea. A phylogenetic study suggested the archaeal origin of the DNA-directed RNA polymerase of Mimivirus (Moreira and Brochier-Armanet, 2008). In addition, two genes of Marseillevirus exhibited best BLAST hits against archaeal genes but this potential relationship were not further validated by phylogenetic analyses (Boyer et al., 2009). Thus, giruses and archaea do not seem to undergo significant lateral exchanges, probably, to some extent, because they share a less sympatric lifestyle. However, these results might be biased by the current paucity of archaeal genomes in sequence databases.

ARVs show more extensive potential LGTs with bacteria. BLAST-based methods identified respectively 96 genes (10%) in Mimivirus, 57 genes (13%) in Marseillevirus, and 7 additional genes in Lausannevirus that may have been exchanged with bacteria (Filee et al., 2008; Boyer et al., 2009; Thomas et al., 2011). Moreover, phylogenetic reconstructions confirmed 29 cases of gene transfer in Mimivirus and 49 in Marseillevirus (Moreira and Brochier-Armanet, 2008; Boyer et al., 2009). The analysis of LGTs in all NCLDVs showed that bigger genomes have a higher propensity to acquire genes from bacteria (La Scola et al., 2010). Interestingly, viruses infecting host that do not graze on bacteria exhibit less genes of potential bacterial origin (La Scola et al., 2010), suggesting that host grazing on microorganisms provide a favorable niche for genetic exchanges.

A few cases of lateral transfers between amoeba-resisting viruses and bacteria were documented. Moreira et al. (Moreira and Brochier-Armanet, 2008) reported the clustering of two Mimivirus genes to *Legionella pneumophila* and *Campylobacter spp.*, two bacterial species able to infect amoebae. Moreover, the dUTPase of Lausannevirus exhibited highest similarity to *Candidatus* Amoebophilus asiaticus, a symbiont of *Acanthamoeba* (Thomas et al., 2011). However, phylogenetic reconstruction did not confirm the clustering of both microorganisms. This may be due to the short length of the protein leaving only few phylogenetically informational sites. Finally, no phylogeny showed a cluster of Marseillevirus and ARBs such as *Legionella* or *Parachlamydia* (Boyer et al., 2009).

#### Viral genome extremities are hotspots for gene exchange

Genes potentially acquired by LGT from bacteria were shown to cluster at the extremities of Mimivirus linear genome, whereas they are more scattered throughout the genomes of other NLCDV (Filee et al., 2007, 2008). On the contrary, NCLDV core genes and genes of eukaryotic origin are centered on the genome sequence. Interestingly, Megavirus and Mimivirus are largely collinear in the central genomic region and exhibit a single inversion and a translocation (Arslan et al., 2011). In *Mimiviridae*, this is not correlated to a decrease in sequence conservation or to an enrichment in transposases in these regions. Similarly, Marseillevirus, and Lausannevirus show only a few inversions between 150 and 350 kb (Thomas et al., 2011). In contrast, the genomic extremities show an almost total loss of colinearity. A similar feature was observed in poxviruses (Esteban and Hutchinson, 2011), and, as suggested by Arslan et al. (Arslan et al., 2011) this might reflect some similarities in the system of genome replication, and an eventual coupling of replication and recombination that would favor the rearrangements, insertion, or deletion of genes at the extremities of the viral chromosomes.

### Extent of genetic exchanges in ARBs

Intracellular bacteria are thought to evolve by genome reduction rather than by acquisition of new genes (Moran, 2002). Individual genome publications as well as more detailed phylogenetic studies explored the events of LGT between bacteria, between bacteria and eukaryotes as well as with other microorganisms. Again, the various methods and cutoffs used make it difficult to directly compare the extent of genetic exchanges in each bacterial phyla. However, they provide an essential knowledge to appreciate the importance of such transfer events and the large diversity in the potential couples of donor-acceptor as shown by some well studied examples in Table 5.

**Table 5:** **Examples of lateral gene transfer with amoeba-resisting bacteria**.

**Gene**	**Function**	**Partners of LGT**	**References**
*legS2*	Sphingosine-1-phosphate lyase	*L. pneumoniae*-Protist	Degtyar et al., 2009
*dhcR7, dwf*	7-dehydrocholesterol reductase	*Legionellae*-Amoeba	Moliner et al., 2009; Thomas and Greub, 2010
*tlc, ntt*	ADP/ATP translocase, Nucleotides transporter	*Chlamydiales-Rickettsiales*-Plants	Greub and Raoult, 2003; Linka et al., 2003; Schmitz-Esser et al., 2004
*ralF*	Sec7 domain-containing protein	Eukaryota*-Legionella-Rickettsia*	Cox et al., 2004
*tra*	Type IV secretion system	Unknown-*Rickettsia Proteobacteria-Chlamydiales*	Gillespie et al., 2010; Greub et al., 2004a

#### Lateral gene transfers with eukaryotes

In *A. asiaticus*, five genes exhibiting typical eukaryotic domains were identified as laterally transferred with eukaryotes (Schmitz-Esser et al., 2010). Two corresponding phylogenies clustered *Amoeobophilus* with the amoeba *D. discoideum*. However, the precise identity of the donor remains unknown due to the limited availability of eukaryotic homologs and especially *Acanthamoeba*, the natural host of this bacterium.

Little evidence was found for recent LGTs in classical *Chlamydia* (Dalevi et al., 2002) and in *Pr. amoebophila* (Horn et al., 2004). Following the analysis of the first *Chlamydiales* genomes (Stephens et al., 1998; Horn et al., 2004), several studies identified plant genes of chlamydial origin and reported the importance of chlamydial genes in the establishment of plant plastid functions (Brinkman et al., 2002; Huang and Gogarten, 2007; Moustafa et al., 2008; Suzuki and Miyagishima, 2010). A recent study including newly sequenced *Chlamydia*-related bacteria demonstrated that 53 genes were transferred from *Chlamydiales* to plants (*n* = 31), to a subgroup of plants (*n* = 7) or in an unknown direction (*n* = 9) (Collingro et al., 2011). These genes encode a variety of functions listed by decreasing importance: carbohydrate metabolism, energy production, lipid metabolism, and translation. The central metabolic functions encoded by these genes support an essential contribution of *Chlamydiales* to plant genomes.

*L. pneumophila* and *L. longbeachae* harbor respectively 30 and 70 proteins with highest similarity to eukaryotic proteins (Cazalet et al., 2004; Kozak et al., 2010). Thomas et al. (Thomas and Greub, 2010) reported that out of 30 eukaryotic-like proteins of *L. pneumophila*, 8 were phylogenetically related to proteins encoded in ongoing amoebal genomes and expressed sequence tags. Interestingly, *L. pneumophila* likely acquired from a protist LegS2, a homolog to the sphingosine-1-phosphate lyase (SPL) that is highly conserved in eukaryotes (Degtyar et al., 2009). This proteins harbor an extra C-terminal domain, absent from its eukaryotic homologs, that is used to trigger its translocation into the host cells using the type IV Icm/Dot secretion system and to target it to the mitochondria. This demonstrates the ability of *Legionella* to alter proteins of eukaryotic origin to better use its host. In addition, Degtyar et al. (2009) denoted that the pattern of presence/absence of effector-encoding genes does not correlate with the *Legionella* phylogenetic tree of the genus, suggesting that these genes were acquired through a massive lateral transfer and lost during evolution.

In a recent study on the occurrence LGTs between *L. drancourtii* and *P. acanthamoebae*, we showed that three proteins of *L. drancourtii* clustered with eukaryotes in phylogenies: a keto acid dehydrogenase, a hypothetical protein and the 7-dehydrocholesterol reductase (Gimenez et al., 2011). This latter was previously shown to be present in *C. burnettii*, two *Chlamydia*-related bacteria (*P. amoebophila* and *P. acanthamoebae*) and Mimivirus but absent from other *Legionellae* (Moliner et al., 2009; Thomas and Greub, 2010). Moliner et al. suggested that the 7-dehydrocholesterol reductase had been acquired by a chlamydial ancestor from viridiplantae and further transferred to other intracellular bacteria. Thank to the availability of sequences from the amoeba *Naegleria gruberi* that clustered with ARBs, Thomas and Greub (Thomas and Greub, 2010) proposed that this gene had been directly exchanged between an amoeba and intracellular bacteria.

Another example of multiple lateral exchanges is the ADP/ATP translocase that is present only in some intracellular bacteria, green plants, and algae plastids (Winkler, 1976; Greub and Raoult, 2003). The first study considered that these genes were transferred from plants to *Rickettsiae* and *Chlamydiae* (Wolf et al., 1999). Subsequently, Amiri et al. (2003) suggested that these genes were of rickettsial origin. However, detailed phylogenetic analyses suggested an early gene duplication in *Chlamydiae*, an exchange between *Chlamydiae* and *Rickettsiae*, and a transfer from *Chlamydiae* to plants (Greub and Raoult, 2003; Linka et al., 2003; Schmitz-Esser et al., 2004). Based on 16S rRNA divergence and fossile estimates, this transfer from *Chlamydiae* to plants was dated to 1 billion years ago (Greub and Raoult, 2003). Interestingly, in *A. asiaticus* the single ADP/ATP translocase is flanked by two nearly identical IS, suggesting it has been acquired by transposon mediated transfer.

#### Lateral gene transfers with prokaryotes

The analysis of *A. asiaticus* genome revealed that 54 genes showed bidirectional best BLAST hits with organisms outside the phylum *Bacteroidetes* (Schmitz-Esser et al., 2010). Among them, 37 had a stable and well supported position in phylogenetic trees indicating they have been acquired laterally. Those represent ancient transfer events whose direction cannot be unambiguously determined. A large marjority of those genes are shared with other amoeba-associated bacteria, and in particular with *Rickettsiae*.

Only few examples of LGTs were reported in the *Chlamydiales.* Based on the presence of conserved indels, three genes were proposed to be exchanged with *Archaea* (*glmU*) and *Actinobacteria* (*murA* and *glyA*), respectively (Griffiths and Gupta, 2002, 2006). Moreover, sets of proteins unique to different chlamydial family or members were investigated by BLAST leading to the discovery of 33 cases of putative gene loss and transfer (Griffiths et al., 2006). In an extensive study of the cross-talk between *P. acanthamoebae* and *L. drancourtii*, 7 genes were likely involved in a direct LGT event (Gimenez et al., 2011). Moreover, 18 tree topologies suggested a transfer from *P. acanthamoebae* to an ancestor of the *Legionellae.* In addition, 4 topologies clustered various members of the *Chlamydiales* and the *Legionellales* indicating probable ancient exchanges between ancestors of these two otherwise distantly related clades.

Coscolla et al. (Coscolla et al., 2011) extensively studied the importance of LGT with other bacteria in the constitution of the *L. pneumophila* pangenome. A significant proportion (18%, *n* = 704) of the 3846 genes forming the pangenome were likely transferred with the following bacterial phyla in decreasing order of importance: *β-proteobacteria* (*n* ≈ 200), α-*proteobacteria* (*n* > 100), *Actinobacteria* (*n* ≈ 100), *Acidobacteria* and *Bacteroidetes*, followed by *Cyanobacteria*, *Firmicutes*, and *Chlamydiales*.

BLAST-based searches of *Rickettsia bellii* proteome highlighted respectively 72 and 22 proteins with a best hit among *Legionellae* and *Parachlamydiaceae* (Ogata et al., 2006). Similar searches with the proteomes of related α-proteobacteria suggested that *R. bellii* and *R. felis* were significantly enriched in sequences homologous to *Legionellae* and *Parachlamydiaceae* (8.8 and 8.2%, respectively) than other bacteria that do not belong to the *Rickettsiales* order (*Pelagibacter ubique*: 2.5%, *Mesorzizobium loti*: 0.9%, *Brucella melitensis, Caulobacter crescentus*). Further BLAST and phylogenetic analyses highlighted respectively 6 and 3 genes likely transferred laterally with *Legionellaceae* and *Parachlamydiaceae*, respectively. Among these is the Sec7 domain-containing protein that is homologous to RalF protein of *L. pneumophila.* This protein is secreted into the host cytosol where it helps recruiting ADP-ribosylation factors to the replicative vacuole (Nagai et al., 2002). Sec7 protein was suggested to be transferred from eukaryotes to bacteria and then in a secondary event between *Legionella* and *Rickettsia* (Cox et al., 2004).

A type four secretion system (T4SS) similar to an F-like conjugation system is encoded by *tra* genes in the genomes of *Rickettisa* (Ogata et al., 2005c, 2006). This system may translocate effectors into the host or mediate DNA transfer among bacteria (Christie, 2001; Ding et al., 2003). Its conservation among *Rickettsiales* and phylogenetic reconstructions suggests an ancestral acquisition of the rickettsial T4SS from organisms that do not belong to α-*proteobacteria* (Gillespie et al., 2010). A highly similar system was identified in several *Chlamydiales;* on a genomic island of *P. amoebophila* (Greub et al., 2004a), as a partial operon in *P. acanthamoebae* (Greub et al., 2009) as well as on the plasmid of *S. negevensis* (Collingro et al., 2011). First proposed to be of proteobacterial origin (Greub et al., 2004a), new genomic information suggested that the T4SS was acquired by an ancestor of the *Chlamydia*-related bacteria and subsequently lost in the *Waddliaceae* family (Collingro et al., 2011). This type IV secretion system likely contributes to the genome evolution of these intracellular pathogens by allowing the formation of conjugative pilus and the transfer of DNA from the donor to the recipient cell.

## Exchanged protein functions and origin of transfer

ARBs and ARVs both share the extremely sympatric lifestyle of ARMs. Amoebae represent a specific niche with particular requirements and strong selection pressure to enable the survival and growth of microorganisms. In this constrained environment, the acquisition and the establishment in the population of particular genes that provide advantages to the resisting organism in the host-pathogen interaction are thus naturally favored. The analysis of both ARBs and ARVs suggested a link between the function of laterally transferred genes and the potential origin of transfer. The functions may be categorized into (i) core functions for replication, transcription, and translation, (ii) metabolic pathways, (iii) mobile elements and systems for DNA conjugation or effector translocation, and (iv) eukaryotic domain of unclear function that may help in interacting with host cell factors.

As microorganisms extremely dependant on the host machinery, giruses have preferentially acquired by horizontal transfer genes belonging to the first and second category compared to bacteria. However, a few examples discussed above underlined their ability to acquire MGEs and to encode several proteins containing eukaryotic domains such as those with ankyrin repeats or leucine-rich repeats (LRRs), which fall within the third and fourth categories. Interestingly, it was denoted both in Marseillevirus and Mimivirus that genes involved in translation were more likely acquired from amoebae (Table 6) (Moreira and Brochier-Armanet, 2008; Boyer et al., 2009). For genes belonging to other aforementioned categories, the established origin of transfer differs in the two viruses (Table 6). In Marseillevirus, those involved in signal transduction were acquired from other eukaryotes whereas defense and repair functions, notably encoded by nucleases, were of bacterial or bacteriophage origin (Boyer et al., 2009). Finally, core metabolic functions, protein and lipid modification or degradation were from mixed bacterial and eukaryotic origin. In Mimivirus, genes for tRNA modification, protein folding and molecular chaperones, lipid metabolism, as well as amino acid metabolism were more likely exchanged with eukaryotes. On the contrary, genes involved in nucleotide or polysaccharide metabolism were of bacterial origin.

**Table 6:** **Function and origin of LGTs in amoeba-resisting viruses**.

**Function**	**Mimivirus**			**Marseillevirus**		
	**E**	**B**	**P**	**E**	**B**	**P**
Translation	x^a^			x^a^		
tRNA modification	x					
Repair					x	x
Defense					x	x
Signal transduction				x		
Polysaccharide metabolism		x		x	x	
Nucleotide metabolism		x		x	x	
Amino acid metabolism	x			x	x	
Protein modification and degradation	x			x	x	
Lipid metabolism	x			x	x	

E, Eukaryote; B, Bacteria; P, Bacteriophage.

a More precisely, acquired from host.

Globally, this suggests the role of LGTs to exchange genes conferring selective advantages to the viruses in diverting their host to their own advantage. The assessment of the potential transfer origin relies too much on the availability of certain types of viral, bacterial, and host genome sequences in public databases. The current incomplete picture of donor-acceptor classification hampers the drawing of further relationships.

All ARBs encodes numerous MGEs as well as conserved system for the translocation of effectors or DNA (Lawley et al., 2003; Juhas et al., 2008). Moreover, a large number of eukaryotic-like proteins and proteins harboring eukaryotic domains were discovered in the genomes of many intracellular bacteria like *C. burnetii* (Seshadri et al., 2003), *Legionellae* (Gomez-Valero et al., 2009; Kozak et al., 2010), *Rickettsia* (Ogata et al., 2005c, 2006), *A. asiaticus, M. avium*, and *F. tularensis* (Schmitz-Esser et al., 2010) suggesting their importance in interacting with the host cell. In *L. pneumophila, R. bellii*, and *R. felis*, they represent respectively 3.5, 6.8, and 4.9% of the gene content. These proteins present eukaryotic domains for host-cell interaction such as Ankyrin, TPR/Sel1, LRR, Serine/Threonine protein kinases as well as other protein–protein interaction domains such as F-box and U-box that may interfere with the host ubiquitin system. F-box proteins were discovered as components of the SCF complex that mediates the ubiquitination of proteins targeted for proteolysis and U-box proteins were identified as ubiquitin-protein ligases (Kipreos and Pagano, 2000; Hatakeyama and Nakayama, 2003).

Little is known on the origin and the evolution of the above-mentioned eukaryotic domains in eukaryotes and their parasites. Convergent evolution might explain some homologies, but several studies pointed the exogenic origin of these genes that have been likely acquired laterally from eukaryotes during evolution. Pioneering studies suggested important functions for some of these domains to modulate the host cell mechanisms and enable the efficient replication of amoeba-resisiting microorganisms. An example is the Serine/Threonine protein kinase of *M. tuberculosis* PknG that was shown to prevent phagosome-lysosome fusion and thus to promote the survival of the bacterium (Walburger et al., 2004). In *L. pneumophila*, Al-Quadan and Kwaik (2011) studied the role of an effector that contains an F-box and two Ankyrin repeats in macrophages and in *D. discoideum* amoeba. This effector is translocated through the Dot/Icm system and functions as a linker to dock polyubiquitinated proteins on the vacuole containing the *Legionella*.

## Conclusions

Intracellular microorganisms have long been considered as specialists with limited genomic repertoires and few genes of exogenic origin. On the contrary, ARVs exhibit the largest genomes in the viral world (Raoult et al., 2004; Arslan et al., 2011). Given the importance of these viruses in the debate about the origin of life and the evolution of viruses, the number of known and sequenced ARVs will undoubtedly enlarge rapidly. Moreover, several ARBs of distant families harbor larger genomes than their closest relative infecting mammalian cells (Moliner et al., 2010). As genomic data on ARMs accumulate, a large panel of evidence substantiates the great activity of these microorganisms in transferring genes laterally. The current sequencing of new unicellular eukaryotes will highlight the genetic exchanges occurring in this setting. Broader and more standardized studies are now required to assess if amoebae represent a niche more favorable to lateral gene exchanges compared to other ecological systems and microbial communities such as biofilms or rhizosphere.

It is now clear that the genomes of amoeba-infecting microorganisms are of composite nature as they harbor genes related to all different kingdom of life. Giant viruses and ARBs had the possibility to exchange genes with eukaryotic organisms as well as with other intracellular microorganisms. The flux of genes in multiple directions enables eukaryote–virus–bacteria interactions. This explicitly indicates the role of amoebae as an evolutionary crib for the emergence of new microorganisms. However, data are still lacking to infer the exact prevalence of host to microorganism or microorganism to host transfers. Similarly, until now only few studies evidenced probable direct LGTs between intra-amoebal microorganisms and the prevalence of ARMs to ARMs transfer is presumably underestimated.

It is only recently that amoebal co-culture has developed more broadly as a tool to isolate new ARMs. The difficulties to grow and isolate ARMs, as well as the propensity to use *Acanthamoeba* strains only for co-culture, largely bias our knowledge on the diversity of ARMs and the extent of genetic exchanges occurring within amoebae. The analysis of the genomes of new amoebae other than *Acanthamoeba* and their resisting microorganisms will enable to address if LGTs occurs at a similar rate in *Acanthamoeba* as in other amoebae. The proximity of ARMs in amoebae certainly helps maintaining a close relationship between their genomes through LGT. In a direct experiment, Saisongkorh et al. (2010) recently suggested that *Bartonella* and *Rhizobium radiobacter* can conjugate and exchange a plasmid when co-cultured in *A. polyphaga*. Similar experiments involving the co-culture of two or more microorganisms within the same amoeba over a varying number of generation followed by the isolation of the microorganisms and their resequencing will surely help evidencing such events of lateral transfer.

Genes laterally transferred belong to two categories; (i) those of known function, mostly involved in core processes that clearly improve the abilities of the microorganism to replicate and spread into new hosts and (ii) those of mostly unknown function such as proteins bearing particular interaction domains that may be used in the corruption of the cell machinery by the intracellular microorganism. Further investigations of laterally acquired genes and mechanistic studies on their function should enhance our knowledge on the mechanisms implicated in host–microbe interactions and the evolutionary history of pathogenesis. In this setting, the chimerism of ARMs may be more related to their lifestyle than to their phylogenetic and evolutionary history. The genome sequencing of new amoebae, and especially *A. castellanii*, will also likely reveal to be strongly influenced by LGTs, thus further challenging our current Darwinian perception of eukaryotic evolution.

### Conflict of interest statement

The authors declare that the research was conducted in the absence of any commercial or financial relationships that could be construed as a potential conflict of interest.
